# Association between preexisting long‐term care needs and in‐hospital mortality and long‐term outcomes in older inpatients with pneumonia: A retrospective cohort study

**DOI:** 10.1002/jgf2.70016

**Published:** 2025-04-11

**Authors:** Jumpei Taniguchi, Hayato Yamana, Yuichiro Matsuo, Yusuke Sasabuchi, Hiroki Matsui, Takahide Kohro, Hideo Yasunaga

**Affiliations:** ^1^ Department of Clinical Epidemiology and Health Economics School of Public Health, The University of Tokyo Tokyo Japan; ^2^ Data Science Center Jichi Medical University Tochigi Japan; ^3^ Department of Real‐World Evidence Graduate School of Medicine, The University of Tokyo Tokyo Japan

**Keywords:** administrative claims database, functional outcome, long‐term care needs, older age, pneumonia

## Abstract

**Background:**

Limited evidence exists regarding the impact of baseline functional and cognitive impairments on the outcomes of patients with pneumonia.

**Methods:**

We used medical and long‐term care administrative databases in a prefecture in Japan that contained care need levels assessed using the national standardized certification system. We identified patients aged ≥65 years who were hospitalized for pneumonia between June 2014 and October 2018. The impairments were classified into four categories based on estimated total daily care time: no care needs, support levels 1–2, care needs level 1 (estimated care time of 25–49 min), care needs level 2–3 (50–89 min), and care needs level 4–5 (≥90 min). The primary outcome was the in‐hospital mortality rate. Secondary outcomes were death and care needs at 6 months and 1 year after admission. We evaluated the outcomes based on care need levels and conducted multivariate analyses adjusting for potential confounders.

**Results:**

A total of 15,537 patients (mean age 83.9 years) were included. The in‐hospital mortality rates for patients with no care needs, support levels 1–2 and care needs level 1, care needs levels 2–3, and care needs levels 4–5 were 10.5%, 15.9%, 21.1%, and 24.7%, respectively. The proportions of patients who died or experienced worsening care needs at 6 months were 43.6%, 60.4%, 60.0%, and 50.2%, respectively. Multivariable analyses demonstrated independent associations of preexisting care needs with both in‐hospital mortality and long‐term outcomes.

**Conclusion:**

Preexisting long‐term care needs are associated with short‐ and long‐term outcomes in older inpatients with pneumonia.

## 1 BACKGROUND

Pneumonia is a common infectious disease among older adults, associated with high morbidity and mortality.^1^ As previously reported, the prognosis of pneumonia worsens with increasing age.^2^ Furthermore, older patients often experience persistent symptoms and functional decline even after successful pneumonia treatment.^3^, ^4^

Existing tools for assessing the severity of pneumonia, such as the CURB‐65 score and Pneumonia Severity Index, are based on demographic characteristics, comorbidities, and clinical variables.^5^, ^6^ However, these indices do not account for factors particularly important in older patients with pneumonia, such as frailty and baseline functional and cognitive impairments. Therefore, limited evidence exists regarding the impact of patient frailty and baseline functional and cognitive impairments on short‐term outcomes in patients with pneumonia.^7^, ^8^, ^9^, ^10^, ^11^ Additionally, few longitudinal studies have investigated the long‐term functional decline after hospital admission for pneumonia.^12^, ^13^ Evaluation of long‐term functional outcomes is necessary for developing long‐term medical plans and efficient resource allocation.

This study investigated in‐hospital mortality and long‐term functional outcomes in older patients with preexisting long‐term care needs after admission for pneumonia. Data were obtained from a large administrative claims database of the Japanese National Health Insurance and Long‐Term Care Insurance systems. Care need levels were assessed using the national standard care need certification system within the long‐term care insurance framework.

## 2 METHODS

### 2.1 Data source

This retrospective cohort study was approved by the Institutional Review Board of Jichi Medical University.

The requirement for informed consent was waived due to the de‐identified nature of the data.

Two administrative databases from Tochigi, one of the 47 prefectures in Japan, were used. The first administrative database contained medical care data from the National Health Insurance and Late Elders' Health Insurance.^14^ The former refers to self‐employed individuals, retirees, and their dependents. The latter applies to all individuals aged ≥75 years. This database includes the following information: age, gender, main diagnoses recorded using the International Classification of Diseases, 10th Revision (ICD‐10) codes, medications, surgical and nonsurgical procedures, and costs. The second administrative database was obtained from the Long‐Term Care Insurance system.^15^ This system is a public and mandatory scheme introduced by the government in 2000.^16^ The insurance targets individuals over 65 years of age as primary insurance candidates and those aged 40–64 years with one of 16 specific diseases (such as end‐stage cancer, Alzheimer's disease, or stroke) as secondary insurance candidates. Eligible candidates can access long‐term care insurance services regardless of income level or the availability of informal family care. Eligibility and care need levels are determined using a national standardized care needs certification system.^17^ The database used in this study included the levels of care needs, types of long‐term care services, and associated costs.

### 2.2 Patients

Patients hospitalized for pneumonia (ICD‐10 codes: J10.x–J18.x and J69.x) between June 2014 and October 2018 were identified using a medical insurance database. We excluded (i) patients aged <65 years, (ii) patients who did not receive antibiotics within 2 days after admission to enhance the validity of pneumonia diagnosis, and (iii) patients with <6 months of recorded data before hospitalization to ensure a sufficient look‐back period for assessing preexisting conditions. All eligible patients were followed up until death, 1 year post‐admission, or withdrawal from medical insurance.

### 2.3 Care needs level

Under the Japanese Long‐Term Care Insurance system, an insured individual or their caregiver must apply at the municipal office to obtain a nationally standardized care needs certification. The application requires basic information about the individual's health status and level of independence in daily activities. Upon receiving the application, the municipality dispatches trained staff to visit the applicant's home or care facility. During this visit, they used a comprehensive 74‐item questionnaire to assess the applicants' physical and mental health and daily living capabilities. The questionnaire covers areas such as physical functions, daily activities, cognitive abilities, behavioral disorders, adjustment to social life, and daily use of medical services. Based on the assessment results, a computer program evaluates the extent and degree of care required to determine the care level. These are categorized into seven levels, ranging from “support level 1” to “care needs level 5,” based on the total daily estimated care minutes per day: support level 1 (25–31 min); support level 2 (32–49 min); care needs level 1 (32–49 min); care needs level 2 (50–69 min); care needs level 3 (70–89 min); care needs level 4 (90–109 min); and care needs level 5 (≥110 min). The findings and calculated care levels are submitted to the Nursing Care Needs Certification Board, which is composed of physicians, nurses, and welfare professionals. The committee reviews the submitted information to finalize care‐level determinations. Care needs levels are generally reassessed at 6‐ to 12‐month intervals. Additional reassessments may be conducted if significant changes occur in the health status of the insured individual or care requirements. Previous studies have shown strong correlations between care needs levels, activities of daily living (as calculated by Barthel Index scores), and cognitive decline.^18^, ^19^, ^20^ A previous study provided details on the questionnaires, certification process, and content of the care services.^21^

In this study, care needs were categorized into the following four groups based on previous studies reporting a relationship between care need levels and Barthel Index scores: no care needs, support level 1–2 and care needs level 1 (comparable to Barthel Index scores of 85–95), care needs level 2–3 (65–80), and care needs level 4–5 (<40).^18^, ^21^

### 2.4 Variables and outcomes

Patient characteristics included age (65–74, 75–84, 85–94, and ≥95 years), gender (male and female), fiscal year of admission (2014, 2015, 2016, 2017, and 2018), Charlson comorbidity index (calculated using ICD‐10 codes during the 6 months before admission), types of antibiotics administered (penicillins, cephalosporins, fluoroquinolones, carbapenems, macrolides, tetracyclines, and anti‐methicillin‐resistant *Staphylococcus aureus* antibiotics), teaching hospital admission, intensive care or high care unit admission, and treatments initiated within 2 days of admission, such as oxygenation, renal replacement therapy, mechanical ventilation, feeding tube placement, and vasopressor use. All baseline patient variables were used as covariates.

The primary outcome measure was mortality during the index hospitalization. Secondary outcome measures included survival and care needs at 6 months and 1 year post‐admission and changes in care needs levels at 6 months and 1 year post‐admission. Secondary outcomes were analyzed as exploratory endpoints, and multiple comparisons were not accounted for in the analysis.

### 2.5 Statistical analysis

We investigated the characteristics and outcomes of patients admitted with pneumonia, stratified by preexisting care need levels. We generated Kaplan–Meier curves, stratified by preexisting care needs, for death until 1 year after the index admission and employed the log‐rank test to evaluate the differences. Death during the first hospitalization, death by 6 months and 1 year, and changes in care needs by 6 months and 1 year were analyzed using multivariable logistic regression models with preexisting care need levels and all baseline patient characteristics, including the fiscal year of admission. Changes in care need levels were compared using odds ratios for “worsening or death” versus “no change or improvement.” Additionally, the care needs levels at 6 months and 1 year after admission were categorized into an ordinal five‐category scale (from best “no care needs” to worst “death”) and evaluated with a multivariable proportional odds model, adjusting for preexisting care needs levels and all baseline patient characteristics. All analyses of outcomes at 6 months or 1 year were conducted after excluding patients without the respective observation periods. For all multivariable models, we excluded patients with missing data and conducted a complete case analysis. Variables in the models were selected based on previous literature and clinical relevance.^21^ All statistical analyses were performed using STATA/SE version 18.0 software (STATA, College Station, TX, USA).

## 3 RESULTS

**FIGURE 1:**
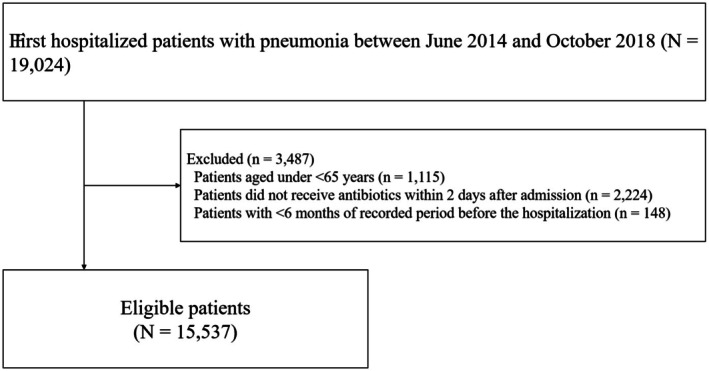
Study design.

A total of 19,024 patients with pneumonia were admitted to our hospital between June 2014 and October 2018. Among these patients, 15,537 met the eligibility criteria (Figure 1). The patient characteristics are presented in Table 1. Two patients (<0.01%) had missing values for the fiscal year. The mean age was 83.9 years (standard deviation: 7.5 years), and 54.8% were male. No significant difference was observed in age among patients with long‐term care needs. However, the proportion of males decreased as their level of long‐term care needs increased. Among patients with long‐term care needs, the use of cephalosporin antibiotics decreased, and the use of ampicillin‐sulbactam and piperacillin‐tazobactam increased as the level of care needs increased. The proportion of patients who received oxygen therapy and feeding tube nutrition also increased with the increasing levels of care required; 88 and 117 patients had observation periods of less than 6 months and 1 year, respectively. The patient characteristics after exclusion are presented in Tables S1 and S2.

**TABLE 1:** Patient characteristics stratified by preexisting care needs before admission.

Variables	Overall (*N* = 15,537)	Preexisting care needs before admission			
		No care needs (*N* = 5357)	Support level 1–2 and care needs level 1 (*N* = 2435)	Care needs level 2–3 (*N* = 3550)	Care needs level 4–5 (*N* = 4195)
Age, years, mean (SD)	83.9 (7.5)	79.9 (7.5)	85.6 (6.3)	86.4 (6.6)	85.8 (7.0)
65–74 years, *n* (%)	2032 (13.1%)	1347 (25.1%)	151 (6.2%)	215 (6.1%)	319 (7.6%)
75–84 years, *n* (%)	5391 (34.7%)	2390 (44.6%)	765 (31.4%)	963 (27.1%)	1273 (30.3%)
85–94 years, *n* (%)	7209 (46.4%)	1541 (28.8%)	1393 (57.2%)	2040 (57.5%)	2235 (53.3%)
≥95 years, *n* (%)	905 (5.8%)	79 (1.5%)	126 (5.2%)	332 (9.4%)	368 (8.8%)
Male, *n* (%)	8520 (54.8%)	3624 (67.7%)	1240 (50.9%)	1798 (50.6%)	1858 (44.3%)
Fiscal year, *n* (%)					
2014	3357 (21.6%)	1175 (21.9%)	457 (18.8%)	716 (20.2%)	1009 (24.1%)
2015	3523 (22.7%)	1240 (23.1%)	561 (23.0%)	779 (21.9%)	943 (22.5%)
2016	3409 (21.9%)	1158 (21.6%)	536 (22.0%)	793 (22.3%)	922 (22.0%)
2017	3420 (22.0%)	1167 (21.8%)	561 (23.0%)	828 (23.3%)	864 (20.6%)
2018	1826 (11.8%)	615 (11.5%)	320 (13.1%)	434 (12.2%)	457 (10.9%)
Missing	2 (0.0%)	2 (0.0%)	0 (0.0%)	0 (0.0%)	0 (0.0%)
Charlson Comorbidities Index, *n* (%)					
0	2755 (17.7%)	1139 (21.3%)	302 (12.4%)	525 (14.8%)	789 (18.8%)
1–2	5887 (37.9%)	1885 (35.2%)	948 (38.9%)	1395 (39.3%)	1659 (39.5%)
3–4	4193 (27.0%)	1362 (25.4%)	672 (27.6%)	1003 (28.3%)	1156 (27.6%)
≥5	2702 (17.4%)	971 (18.1%)	513 (21.1%)	627 (17.7%)	591 (14.1%)
Types of antibiotics, *n* (%)					
Penicillins					
Benzylpenicillin	704 (4.5%)	260 (4.9%)	120 (4.9%)	147 (4.1%)	177 (4.2%)
Ampicillin‐Sulbactam	5324 (34.3%)	1609 (30.0%)	799 (32.8%)	1289 (36.3%)	1627 (38.8%)
Piperacillin‐Tazobactam	3124 (20.1%)	1041 (19.4%)	468 (19.2%)	679 (19.1%)	936 (22.3%)
Cephalosporins	5798 (37.3%)	2230 (41.6%)	987 (40.5%)	1283 (36.1%)	1308 (31.2%)
Fluoroquinolones	859 (5.5%)	388 (7.2%)	119 (4.9%)	162 (4.6%)	190 (4.5%)
Carbapenems	1101 (7.1%)	391 (7.3%)	151 (6.2%)	245 (6.9%)	314 (7.5%)
Macrolides	910 (5.9%)	523 (9.8%)	166 (6.8%)	127 (3.6%)	94 (2.2%)
Tetracyclines	630 (4.1%)	321 (6.0%)	90 (3.7%)	99 (2.8%)	120 (2.9%)
Anti‐MRSA antibiotics	237 (1.5%)	131 (2.4%)	38 (1.6%)	31 (0.9%)	37 (0.9%)
Teaching hospital admission, *n* (%)	446 (2.9%)	131 (2.4%)	68 (2.8%)	119 (3.4%)	128 (3.1%)
ICU or HCU admission, *n* (%)	203 (1.3%)	80 (1.5%)	43 (1.8%)	33 (0.9%)	47 (1.1%)
Treatment within 2 days of admission day, *n* (%)					
Oxygenation	9280 (59.7%)	2971 (55.5%)	1451 (59.6%)	2186 (61.6%)	2672 (63.7%)
Renal replacement therapy	167 (1.1%)	75 (1.4%)	36 (1.5%)	32 (0.9%)	24 (0.6%)
Mechanical ventilation	660 (4.2%)	286 (5.3%)	120 (4.9%)	111 (3.1%)	143 (3.4%)
Feeding tube	288 (1.9%)	47 (0.9%)	14 (0.6%)	38 (1.1%)	189 (4.5%)
Vasopressor	270 (1.7%)	115 (2.1%)	40 (1.6%)	43 (1.2%)	72 (1.7%)

*Note*: The total number of etiologies does not add up to 100% as more than one cause can be assigned to a single patient.

Abbreviations: MRSA, methicillin‐resistant *Staphylococcus aureus*; SD, standard deviation.

**FIGURE 2:**
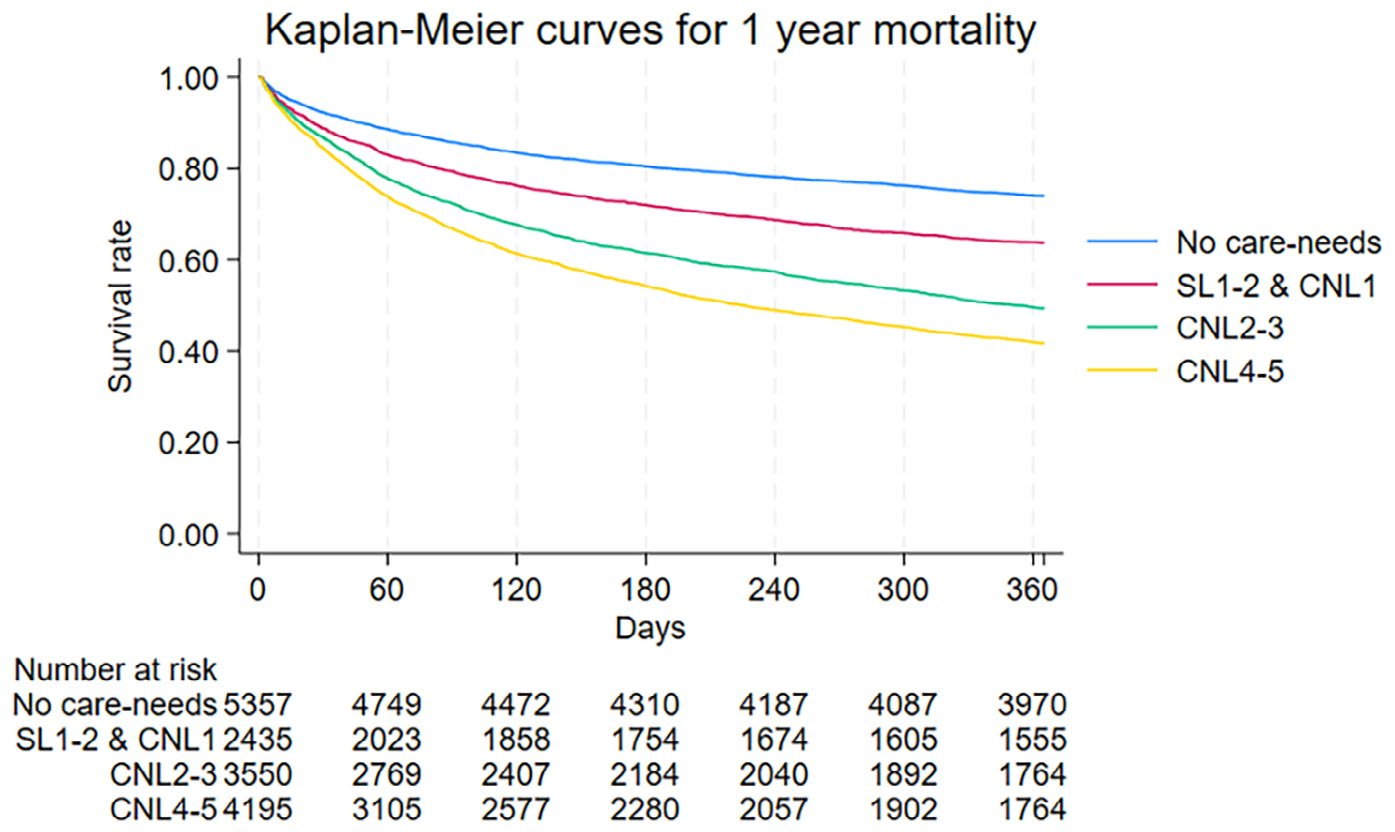
Kaplan–Meier curves for 1‐year mortality after first admission stratified by preexisting care needs levels. CNL, care needs level; SL, support level.

Patient outcomes are presented in Tables 2 and Tables S3 and S4. The overall mortality rate at first admission was 17.6% (2737/15,537). The proportions were 10.5%, 15.9%, 21.1%, and 24.7% in patients with no care needs, support level 1–2 and care needs level 1, care needs level 2–3, and care needs level 4–5, respectively (Table 2). A similar increasing trend was observed in mortality at 6 months and 1 year after hospitalization (Tables S3 and S4). The proportions of patients with no care needs, support levels 1–2 and care needs levels 1, 2–3, and 4–5 who experienced a worsened care needs level at 6 months after their first admission were 23.9%, 32.2%, 21.2%, and 4.0%, respectively, and when including deaths, these proportions were 43.6%, 60.4%, 60.0%, and 50.2%, respectively (Table S3). A few patients experienced improved care needs levels 6 months after the first admission. A similar trend was observed 1 year after hospitalization (Table S4). Figure 2 presents the Kaplan–Meier curves following index hospitalization. Survival time varied significantly according to preexisting care needs (*p* < 0.001).

**TABLE 2:** Primary outcome stratified by preexisting care need at the admission.

Variables	Overall (*N* = 15,537)	Preexisting care needs before admission			
		No care needs (*N* = 5357)	Support level 1–2 and care needs level 1 (*N* = 2435)	Care needs level 2–3 (*N* = 3550)	Care needs level 4–5 (*N* = 4195)
In‐hospital death, *n* (%)	2737 (17.6%)	561 (10.5%)	388 (15.9%)	750 (21.1%)	1038 (24.7%)

Table 3 presents the results of the multivariate analyses. The odds of death during the first hospitalization increased in those with higher preexisting care needs levels: odds ratio [OR] of 1.48 (95% confidence interval [CI], 1.28–1.72) for support level 1–2 and care needs level 1, 2.12 (95% CI, 1.86–2.41) for care needs level 2–3, and 2.67 (95% CI, 2.36–3.02) for care needs level 4–5. The analysis of deaths at 6 months and 1 year showed similar results. The odds of worsening care needs levels were pronounced in patients with lower care needs levels, with odds ratios of 1.61 (95% CI, 1.45–1.79) for support level 1–2 and care needs level 1, 1.51 (95% CI, 1.38–1.66) for those with care needs level 2–3, and 1.02 (95% CI, 0.93–1.12) for care needs level 4–5. Analysis of the change in care needs levels at 1 year showed similar results. In the multivariable proportional odds analysis, the care needs levels at 6 months after first admission were higher in patients with support level 1–2 and care needs level 1 (OR, 4.01; 95% CI, 3.62–4.45), care needs level 2–3 (OR, 8.87; 95% CI, 7.99–9.85), and care needs level 4–5 (OR, 18.29; 95% CI, 16.42–20.36) compared to patients with no care needs before the first admission.

**TABLE 3:** Analysis of the associations between preexisting care needs before admission and the outcomes.

Outcomes	Preexisting care needs before admission			
	No care needs	Support level 1–2 and care needs level 1	Care needs level 2–3	Care needs level 4–5
Primary outcome				
In‐hospital death, OR (95% CI)	Reference	1.48 (1.28–1.72)	2.12 (1.86–2.41)	2.67 (2.36–3.02)
Secondary outcome				
Outcome at 6 months from admission				
Death at 6 months from admission, OR (95% CI)	Reference	1.43 (1.27–1.60)	2.32 (2.09–2.58)	3.33 (3.01–3.68)
Changes in care needs at 6 months from admission, OR (95% CI)	Reference	1.61 (1.45–1.79)	1.51 (1.38–1.66)	1.02 (0.93–1.12)
Care needs at 6 months from admission, OR (95% CI)	Reference	4.01 (3.62–4.45)	8.87 (7.99–9.85)	18.29 (16.42–20.36)
Outcome at 1 year from admission				
Death at 1 year from admission, OR (95% CI)	Reference	1.46 (1.25–1.63)	2.67 (2.42–2.94)	3.90 (3.54–4.29)
Changes in care needs at 1 year from admission, OR (95% CI)	Reference	2.05 (1.84–2.29)	2.28 (2.06–2.51)	1.44 (1.32–1.58)
Care needs at 1 year from admission, OR (95% CI)	Reference	3.26 (2.95–3.59)	7.03 (6.39–7.75)	11.76 (10.46–12.97)

Abbreviations: CI, confidence interval; OR, odds ratio.

## 4 DISCUSSION

This population‐based cohort study revealed two important findings. First, we observed an association between preexisting care needs and in‐hospital mortality after adjusting for measured confounders. Second, preexisting care needs were associated with long‐term functional outcomes. Among patients with preexisting support or care needs, 43.6%–60.4% died or had worsened care needs levels 6 months after admission. These findings indicate the importance of incorporating baseline functional and cognitive impairments, alongside pneumonia severity, to accurately predict short‐ and long‐term outcomes following hospitalization for pneumonia. Furthermore, hospitalization for pneumonia may exacerbate functional decline or death in patients requiring support or care.

Our study has two main strengths. First, we used a long‐term care needs assessment based on the time required for care and investigated its association with the short‐term outcomes of pneumonia. The level of long‐term care needs has been reported to be associated with patient frailty and multimorbidity, highlighting the considerable vulnerability of this population following acute illnesses.^18^, ^21^, ^22^ This vulnerability may be explained by several mechanisms, including reduced physiological reserve, impaired immune function, poor nutritional status, and a limited ability to recover from acute illness.^4^, ^7^, ^8^, ^9^, ^10^, ^11^, ^12^ Additionally, patients with higher care needs usually have cognitive impairment and functional dependency, which may contribute to delayed diagnosis, more complex treatment, and hindered recovery. Previous studies have used the Barthel Index or the Clinical Frailty Scale to evaluate the relationship between baseline functional impairment and short‐term outcomes of acute illnesses.^23^, ^24^ However, while these indices are useful for rapidly identifying individuals at risk of frailty, they primarily assess physical function at a single point and rely heavily on the subjective judgment of the evaluator, raising concerns about the consistency of the evaluation. In contrast, our long‐term care needs assessment integrates objective evaluations through computer algorithms using a comprehensive 74‐item questionnaire, evaluations by experienced administrative personnel, and inputs from multiple medical professionals. This offers a reliable indicator of functional and cognitive impairment in older adults. Furthermore, while the CURB‐65 score, a frequently used pneumonia severity index, predicts short‐term outcomes, a recent study reported its suboptimal performance in older adults.^25^ Therefore, future research integrating pneumonia severity indices and the long‐term care needs assessment may enable more accurate evaluation of short‐term outcomes in this population. Second, we examined the long‐term outcomes of functional decline and death using a large administrative claims database. Previous studies examining functional decline after hospitalization for pneumonia were limited to a short‐term follow‐up of 2 months or have relied on subjective outcome measures, such as telephone interviews or questionnaires from patients or their caregivers.^7^, ^13^ We measured objective and valid long‐term outcomes using data from the public long‐term care insurance system. Our results showed that approximately 50% of patients with support or care needs died or had a worsening level of care within 6 months of admission, highlighting the poor long‐term prognosis of this population. This finding supports the need for medical professionals to consider physical and cognitive decline, as reflected by preexisting long‐term care needs when managing acute disease admissions in older patients. For example, identifying functional impairments may help pinpoint patients at higher risk of adverse clinical outcomes. Targeted strategies, such as a more intensive follow‐up, comprehensive discharge planning, nutritional support, rehabilitation, and the use of post‐acute care services, may also be beneficial for improving long‐term prognosis in this vulnerable population. Additionally, future health service research is needed to examine how care interventions based on this indicator improve long‐term patient outcomes.

### 4.1 Limitations

This study had some limitations. First, there may have been individuals who were dependent on activities of daily living at the time of hospitalization but did not receive care services. Therefore, there may be misclassification of care need levels at the time of admission. Individuals who actually required care may have been classified as having no care needs, which could have biased the results toward worsening the outcomes of the no care needs group. Second, the long‐term care insurance systems differ across countries. The generalizability of our results is limited. However, previous studies have reported a strong correlation between care need levels, the Barthel index, and cognitive function.^18^, ^19^, ^20^ Moreover, similar care needs scores, based on the time required for support and care, have been used in other countries.^26^, ^27^ Third, this study did not consider post‐discharge events after the first admission. Long‐term functional outcomes may have been influenced by post‐discharge events. Fourth, the in‐hospital mortality rate in our study was 17.6%, higher than that reported in previous studies.^28^, ^29^ Additionally, the proportion of patients admitted to teaching hospitals was low. Our study population may have included a higher proportion of vulnerable patients from regions with limited medical resources. Furthermore, differences in the databases used across studies may help explain the variation in results. These factors may further limit the generalizability of our findings.

## 5 CONCLUSION

This retrospective observational study of older inpatients with pneumonia showed worse short‐ and long‐term outcomes in those with preexisting long‐term care needs. Functional and cognitive status, as expressed by care need levels, may be useful in predicting prognosis.

## AUTHOR CONTRIBUTIONS

**Jumpei Taniguchi:** Conceptualization; methodology; data curation; investigation; formal analysis; supervision; visualization; project administration; writing – original draft; writing – review and editing; validation. **Hayato Yamana:** Methodology; software; investigation; supervision; project administration; writing – original draft; writing – review and editing; resources. **Yuichiro Matsuo:** Data curation; formal analysis; project administration; writing – review and editing. **Yusuke Sasabuchi:** Resources; supervision; project administration; writing – review and editing. **Hiroki Matsui:** Supervision; resources; project administration; writing – review and editing. **Takahide Kohro:** Software; resources; writing – review and editing; funding acquisition. **Hideo Yasunaga:** Software; supervision; resources; funding acquisition; writing – review and editing; project administration.

## FUNDING INFORMATION

This work was supported by the “Cross‐ministerial Strategic Innovation Promotion Program (SIP) on ‘Integrated Health Care System’” (grant number JPJ012425) and a grant from the The Ministry of Health, Labour and Welfare, Japan (grant number 23AA2003).

## CONFLICT OF INTEREST STATEMENT

The authors have stated explicitly that there are no conflicts of interest in connection with this article.

## ETHICS STATEMENT

Ethics approval statement: This study was approved by the Institutional Review Board of the Jichi Medical University.

Patient consent statement: The board waived the need for informed consent as this was a retrospective study and the anonymous nature of the data.

Clinical trial registration: None.

## Supporting information


Tables S1–S4

## Data Availability

The data are not publicly available.
