# Supplementary material for: Association between preexisting long‐term care needs and in‐hospital mortality and long‐term outcomes in older inpatients with pneumonia: A retrospective cohort study
Source: J Gen Fam Med. 2025 Apr 11;26(4):326–33. doi: 10.1002/jgf2.70016 (PMC12237830; doi:10.1002/jgf2.70016)
Supplement: Supplementary file 1 — Tables S1–S4 [file JGF2-26-326-s001.docx]

**Supplementary Table 1. Patient characteristics after excluding those who dropped out at the 6-month stratified by preexisting care-needs before admission**

| Variables | Overall  (N = 15,449) | Preexisting care-needs before admission | | | |
| --- | --- | --- | --- | --- | --- |
|  |  | No care-needs  (N = 5,329) | Support level 1–2 and care-needs level 1  (N = 2,428) | Care-needs level 2–3  (N = 3,532) | Care-needs level 4–5  (N = 4,160) |
| Age, years, mean (SD) | 83.9 (7.5) | 79.9 (7.5) | 85.6 (6.3) | 86.4 (6.6) | 85.9 (6.9) |
| 65–74 years, n (%) | 2,006 (13.0%) | 1,336 (25.1%) | 149 (6.1%) | 211 (6.0%) | 310 (7.5%) |
| 75–84 years, n (%) | 5,366 (34.7%) | 2,383 (44.7%) | 765 (31.5%) | 958 (27.1%) | 1,260 (30.3%) |
| 85–94 years, n (%) | 7,177 (46.5%) | 1,532 (28.7%) | 1,388 (57.2%) | 2,033 (57.6%) | 2,224 (53.5%) |
| ≥ 95 years, n (%) | 900 (5.8%) | 78 (1.5%) | 126 (5.2%) | 330 (9.3%) | 366 (8.8%) |
| Male, n (%) | 8,476 (54.9%) | 3,608 (67.7%) | 1,236 (50.9%) | 1,790 (50.7%) | 1,842 (44.3%) |
| Fiscal year, n (%) |  |  |  |  |  |
| 2014 | 3,340 (21.6%) | 1,171 (22.0%) | 455 (18.7%) | 712 (20.2%) | 1,002 (24.1%) |
| 2015 | 3,500 (22.7%) | 1,233 (23.1%) | 560 (23.1%) | 774 (21.9%) | 933 (22.4%) |
| 2016 | 3,389 (21.9%) | 1,148 (21.5%) | 536 (22.1%) | 790 (22.4%) | 915 (22.0%) |
| 2017 | 3,399 (22.0%) | 1,162 (21.8%) | 558 (23.0%) | 825 (23.4%) | 854 (20.5%) |
| 2018 | 1,819 (11.8%) | 613 (11.5%) | 319 (13.1%) | 431 (12.2%) | 456 (11.0%) |
| Charlson Comorbidities index, n (%) |  |  |  |  |  |
| 0 | 2,728 (17.7%) | 1,124 (21.1%) | 302 (12.4%) | 520 (14.7%) | 782 (18.8%) |
| 1–2 | 5,859 (37.9%) | 1,880 (35.3%) | 945 (38.9%) | 1,389 (39.3%) | 1,645 (39.5%) |
| 3–4 | 4,174 (27.0%) | 1,358 (25.5%) | 669 (27.6%) | 999 (28.3%) | 1,148 (27.6%) |
| ≥ 5 | 2,688 (17.4%) | 967 (18.1%) | 512 (21.1%) | 624 (17.7%) | 585 (14.1%) |
| Types of antibiotics, n (%) |  |  |  |  |  |
| Penicillins |  |  |  |  |  |
| Benzylpenicillin | 700 (4.5%) | 259 (4.9%) | 120 (4.9%) | 145 (4.1%) | 176 (4.2%) |
| Ampicillin-Sulbactam | 5,287 (34.2%) | 1,598 (30.0%) | 798 (32.9%) | 1,283 (36.3%) | 1,608 (38.7%) |
| Piperacillin-Tazobactam | 3,113 (20.2%) | 1,038 (19.5%) | 468 (19.3%) | 676 (19.1%) | 931 (22.4%) |
| Cephalosporins | 5,760 (37.3%) | 2,207 (41.4%) | 981 (40.4%) | 1,276 (36.1%) | 1,296 (31.2%) |
| Fluoroquinolones | 856 (5.5%) | 385 (7.2%) | 119 (4.9%) | 162 (4.6%) | 190 (4.6%) |
| Carbapenems | 1,093 (7.1%) | 386 (7.2%) | 150 (6.2%) | 244 (6.9%) | 313 (7.5%) |
| Macrolides | 906 (5.9%) | 520 (9.8%) | 166 (6.8%) | 126 (3.6%) | 94 (2.3%) |
| Tetracyclines | 623 (4.0%) | 318 (6.0%) | 90 (3.7%) | 96 (2.7%) | 119 (2.9%) |
| Anti-MRSA antibiotics | 236 (1.5%) | 130 (2.4%) | 38 (1.6%) | 31 (0.9%) | 37 (0.9%) |
| Teaching hospital admission, n (%) | 443 (2.9%) | 130 (2.4%) | 67 (2.8%) | 118 (3.3%) | 128 (3.1%) |
| ICU or HCU admission, n (%) | 202 (1.3%) | 79 (1.5%) | 43 (1.8%) | 33 (0.9%) | 47 (1.1%) |
| Treatment within 2 d of admission day, n (%) |  |  |  |  |  |
| Oxygenation | 9,223 (59.7%) | 2,953 (55.4%) | 1,446 (59.6%) | 2,175 (61.6%) | 2,649 (63.7%) |
| Renal replacement therapy | 167 (1.1%) | 75 (1.4%) | 36 (1.5%) | 32 (0.9%) | 24 (0.6%) |
| Mechanical ventilation | 655 (4.2%) | 283 (5.3%) | 120 (4.9%) | 110 (3.1%) | 142 (3.4%) |
| Feeding tube | 285 (1.8%) | 46 (0.9%) | 14 (0.6%) | 38 (1.1%) | 187 (4.5%) |
| Vasopressor | 266 (1.7%) | 115 (2.2%) | 40 (1.6%) | 42 (1.2%) | 69 (1.7%) |

**Supplementary Table 2. Patient characteristics after excluding those who dropped out at the 1 year stratified by preexisting care-needs before admission**

| Variables | Overall  (N = 15,420) | Preexisting care-needs before admission | | | |
| --- | --- | --- | --- | --- | --- |
|  |  | No care-needs  (N = 5,316) | Support level 1–2 and care-needs level 1  (N = 2,419) | Care-needs level 2–3  (N = 3,529) | Care-needs level 4–5  (N = 4,156) |
| Age, years, mean (SD) | 83.9 (7.5) | 80.0 (7.4) | 85.6 (6.3) | 86.4 (6.6) | 85.9 (6.9) |
| 65–74 years, n (%) | 1,995 (12.9%) | 1,327 (25.0%) | 148 (6.1%) | 211 (6.0%) | 309 (7.4%) |
| 75–84 years, n (%) | 5,358 (34.7%) | 2,380 (44.8%) | 761 (31.5%) | 958 (27.1%) | 1,259 (30.3%) |
| 85–94 years, n (%) | 7,168 (46.5%) | 1,531 (28.8%) | 1,385 (57.3%) | 2,030 (57.5%) | 2,222 (53.5%) |
| ≥ 95 years, n (%) | 899 (5.8%) | 78 (1.5%) | 125 (5.2%) | 330 (9.4%) | 366 (8.8%) |
| Male, n (%) | 8,466 (54.9%) | 3,602 (67.8%) | 1,235 (51.1%) | 1,788 (50.7%) | 1,841 (44.3%) |
| Fiscal year, n (%) |  |  |  |  |  |
| 2014 | 3,336 (21.6%) | 1,169 (22.0%) | 454 (18.8%) | 712 (20.2%) | 1,001 (24.1%) |
| 2015 | 3,495 (22.7%) | 1,231 (23.2%) | 558 (23.1%) | 773 (21.9%) | 933 (22.4%) |
| 2016 | 3,379 (21.9%) | 1,145 (21.5%) | 533 (22.0%) | 789 (22.4%) | 912 (21.9%) |
| 2017 | 3,395 (22.0%) | 1,160 (21.8%) | 557 (23.0%) | 824 (23.3%) | 854 (20.5%) |
| 2018 | 1,813 (11.8%) | 609 (11.5%) | 317 (13.1%) | 431 (12.2%) | 456 (11.0%) |
| Charlson Comorbidities index, n (%) |  |  |  |  |  |
| 0 | 2,723 (17.7%) | 1,120 (21.1%) | 301 (12.4%) | 520 (14.7%) | 782 (18.8%) |
| 1–2 | 5,842 (37.9%) | 1,874 (35.3%) | 940 (38.9%) | 1,386 (39.3%) | 1,642 (39.5%) |
| 3–4 | 4,170 (27.0%) | 1,357 (25.5%) | 667 (27.6%) | 999 (28.3%) | 1,147 (27.6%) |
| ≥ 5 | 2,685 (17.4%) | 965 (18.2%) | 511 (21.1%) | 624 (17.7%) | 585 (14.1%) |
| Types of antibiotics, n (%) |  |  |  |  |  |
| Penicillins |  |  |  |  |  |
| Benzylpenicillin | 696 (4.5%) | 257 (4.8%) | 120 (5.0%) | 144 (4.1%) | 175 (4.2%) |
| Ampicillin-Sulbactam | 5,278 (34.2%) | 1,596 (30.0%) | 793 (32.8%) | 1,283 (36.4%) | 1,606 (38.6%) |
| Piperacillin-Tazobactam | 3,108 (20.2%) | 1,036 (19.5%) | 468 (19.3%) | 674 (19.1%) | 930 (22.4%) |
| Cephalosporins | 5,751 (37.3%) | 2,203 (41.4%) | 976 (40.3%) | 1,276 (36.2%) | 1,296 (31.2%) |
| Fluoroquinolones | 853 (5.5%) | 382 (7.2%) | 119 (4.9%) | 162 (4.6%) | 190 (4.6%) |
| Carbapenems | 1,091 (7.1%) | 384 (7.2%) | 150 (6.2%) | 244 (6.9%) | 313 (7.5%) |
| Macrolides | 904 (5.9%) | 519 (9.8%) | 165 (6.8%) | 126 (3.6%) | 94 (2.3%) |
| Tetracyclines | 622 (4.0%) | 318 (6.0%) | 90 (3.7%) | 96 (2.7%) | 118 (2.8%) |
| Anti-MRSA antibiotics | 235 (1.5%) | 129 (2.4%) | 38 (1.6%) | 31 (0.9%) | 37 (0.9%) |
| Teaching hospital admission, n (%) | 442 (2.9%) | 129 (2.4%) | 67 (2.8%) | 118 (3.3%) | 128 (3.1%) |
| ICU or HCU admission, n (%) | 202 (1.3%) | 79 (1.5%) | 43 (1.8%) | 33 (0.9%) | 47 (1.1%) |
| Treatment within 2 d of admission day, n (%) |  |  |  |  |  |
| Oxygenation | 9,208 (59.7%) | 2,945 (55.4%) | 1,442 (59.6%) | 2,173 (61.6%) | 2,648 (63.7%) |
| Renal replacement therapy | 167 (1.1%) | 75 (1.4%) | 36 (1.5%) | 32 (0.9%) | 24 (0.6%) |
| Mechanical ventilation | 653 (4.2%) | 281 (5.3%) | 120 (5.0%) | 110 (3.1%) | 142 (3.4%) |
| Feeding tube | 283 (1.8%) | 46 (0.9%) | 14 (0.6%) | 38 (1.1%) | 185 (4.4%) |
| Vasopressor | 265 (1.7%) | 114 (2.1%) | 40 (1.7%) | 42 (1.2%) | 69 (1.7%) |

**Supplementary Table 3. Secondary outcome at 6 months after admission stratified by preexisting care need at the admission**

| Variables | Overall  (N = 15,449) | Preexisting care-needs before admission | | | |
| --- | --- | --- | --- | --- | --- |
|  |  | No care-needs  (N = 5,329) | Support level 1–2 and care-needs level 1  (N = 2,428) | Care-needs level 2–3  (N = 3,532) | Care-needs level 4–5  (N = 4,160) |
| Care-needs at 6 months after admission, n (%) |  |  |  |  |  |
| No care-needs | 3,110 (20.1%) | 3,007 (56.4%) | 40 (1.6%) | 26 (0.7%) | 37 (0.9%) |
| Support level 1–2 and care-needs level 1 | 1,605 (10.4%) | 515 (9.7%) | 1,038 (42.8%) | 40 (1.1%) | 12 (0.3%) |
| Care-needs level 2–3 | 2,187 (14.2%) | 359 (6.7%) | 325 (13.4%) | 1,462 (41.4%) | 41 (1.0%) |
| Care-needs level 4–5 | 3,517 (22.8%) | 397 (7.4%) | 340 (14.0%) | 634 (18.0%) | 2,146 (51.6%) |
| Death | 5,030 (32.6%) | 1,051 (19.7%) | 685 (28.2%) | 1,370 (38.8%) | 1,924 (46.3%) |
| Changes in care-needs at 6 months after admission, n (%) |  |  |  |  |  |
| Improved | 271 (1.8%) | 0 (0.0%) | 68 (2.8%) | 83 (2.3%) | 120 (2.9%) |
| No change | 7,180 (46.5%) | 3,007 (56.4%) | 893 (36.8%) | 1,330 (37.7%) | 1,950 (46.9%) |
| Worsened | 2,968 (19.2%) | 1,271 (23.9%) | 782 (32.2%) | 749 (21.2%) | 166 (4.0%) |
| Death | 5,030 (32.6%) | 1,051 (19.7%) | 685 (28.2%) | 1,370 (38.8%) | 1,924 (46.3%) |

**Supplementary Table 4. Secondary outcome at 1 year after admission stratified by preexisting care need at the admission**

| Variables | Overall  (N = 15,420) | Preexisting care-needs before admission | | | |
| --- | --- | --- | --- | --- | --- |
|  |  | No care-needs  (N = 5,316) | Support level 1–2 and care-needs level 1  (N = 2,419) | Care-needs level 2–3  (N = 3,529) | Care-needs level 4–5  (N = 4,156) |
| Care-needs at 1 year after admission, n (%) |  |  |  |  |  |
| No care-needs | 2,862 (18.6%) | 2,690 (50.6%) | 59 (2.4%) | 35 (1.0%) | 78 (1.9%) |
| Support level 1–2 and care-needs level 1 | 1,413 (9.2%) | 517 (9.7%) | 785 (32.5%) | 79 (2.2%) | 32 (0.8%) |
| Care-needs level 2–3 | 1,803 (11.7%) | 364 (6.8%) | 386 (16.0%) | 959 (27.2%) | 94 (2.3%) |
| Care-needs level 4–5 | 2,834 (18.4%) | 350 (6.6%) | 304 (12.6%) | 661 (18.7%) | 1,519 (36.5%) |
| Death | 6,508 (42.2%) | 1,395 (26.2%) | 885 (36.6%) | 1,795 (50.9%) | 2,433 (58.5%) |
| Changes in care-needs at 1 year after admission, n (%) |  |  |  |  |  |
| Improved | 525 (3.4%) | 0 (0.0%) | 110 (4.5%) | 149 (4.2%) | 266 (6.4%) |
| No change | 5,260 (34.1%) | 2,690 (50.6%) | 585 (24.2%) | 763 (21.6%) | 1,222 (29.4%) |
| Worsened | 3,127 (20.3%) | 1,231 (23.2%) | 839 (34.7%) | 822 (23.3%) | 235 (5.7%) |
| Death | 6,508 (42.2%) | 1,395 (26.2%) | 885 (36.6%) | 1,795 (50.9%) | 2,433 (58.5%) |
